# Supplementary material for: Multifunctional lithium niobate platform for photodetection and photoacoustic and thermoelastic gas sensing
Source: Nat Commun. 2026 Feb 3;17:2296. doi: 10.1038/s41467-026-69042-7 (PMC12976070; doi:10.1038/s41467-026-69042-7)
Supplement: Supplementary file 1 — Supplementary Information [file 41467_2026_69042_MOESM1_ESM.pdf]

Supplementary Information for

Multifunctional lithium niobate platform for photodetection and photoacoustic and thermoelastic gas sensing

Haoyang Lin[1], Huadan Zheng[1,*], Wenguo Zhu[1], Yongchun Zhong[1], Jianhui Yu[1,*], Hongpeng Wu[2], Zhiwei Jia[3], Jinchuan Zhang[3], Angelo Sampaolo[2,4], Pietro Patimisco[2,4], Huihui Lu[1], Xiaojun Jia[2], Vincenzo Spagnolo[2,4], and Lei Dong[2,*]

[1]Key Laboratory of Optoelectronic Information and Sensing Technologies of Guangdong Higher Education Institutes, Department of Optoelectronic Engineering, Jinan University, Guangzhou, 510632, China

[2]State Key Laboratory of Quantum Optics and Quantum Optics Devices, Institute of Laser Spectroscopy, Shanxi University, Taiyuan, 030006, China

[3]Laboratory of Solid-State Optoelectronics Information Technology, Institute of Semiconductors, Chinese Academy of Sciences, Beijing, China.

[4]PolySense Lab—Dipartimento Interateneo di Fisica, University and Politecnico of Bari, Bari, Italy

*Corresponding author: zhenghuadan@jnu.edu.cn, jianhuiyu@jnu.edu.cn, and donglei@sxu.edu.cn

Supplementary Figures

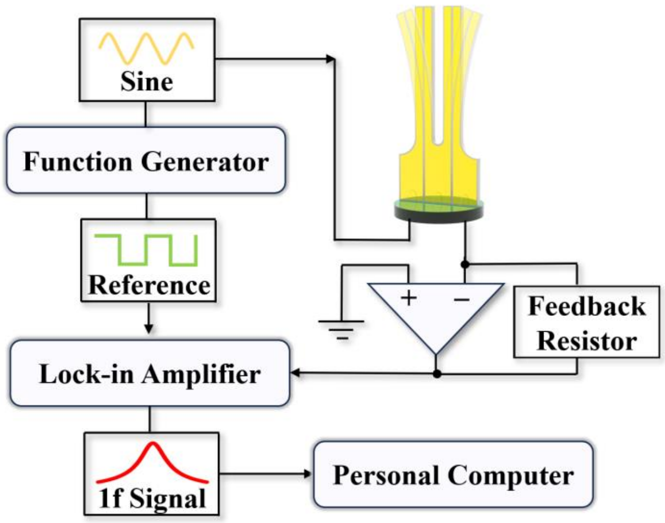

Supplementary Fig. 1 | Experimental setup for resonance frequency measurement of the LN-MFP. Schematic diagram of the measurement circuit used to characterize the resonance frequency of the lithium niobate multifunctional platform (LN-MFP). A function generator outputs a sine-wave excitation to drive the device, while also providing a reference signal to the lock-in amplifier. The piezoelectric response of the LN-MFP is converted to voltage via a transimpedance amplifier with a feedback resistor. The lock-in amplifier extracts the 1f component of the signal, which is recorded by a personal computer for frequency response analysis.

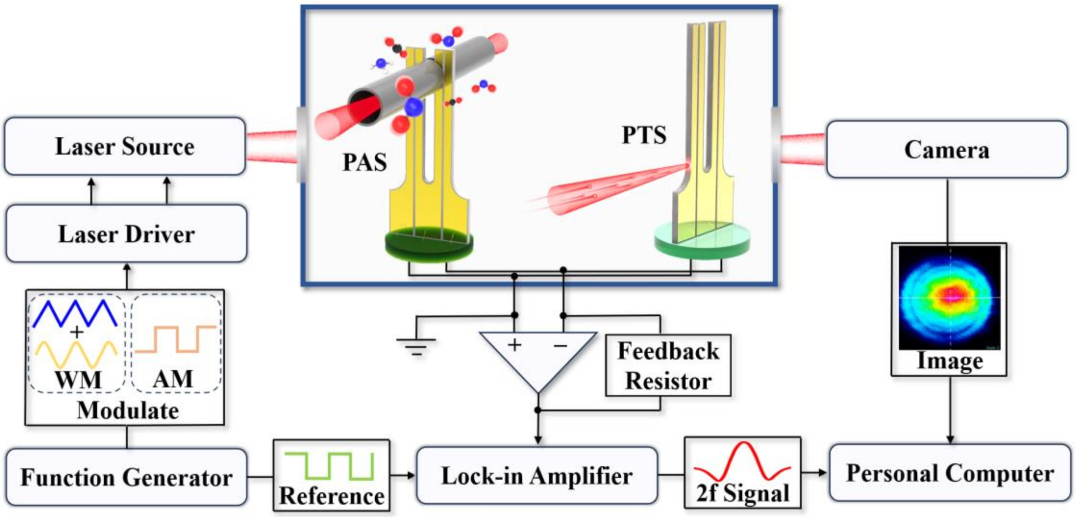

Supplementary Fig. 2 | Schematic of the experimental setup for PAS and LITES using the LN-MFP. Diagram of the integrated setup for photoacoustic spectroscopy (PAS) and light-induced thermoelastic spectroscopy (LITES) using the lithium niobate multifunctional platform (LN-MFP). A function generator provides wavelength and amplitude modulation signals to drive the laser source via a laser driver. The modulated laser beam interacts with target gas and the LN-MFP device in two modes: PAS (acoustic vibration via gas absorption) and PTS (thermoelastic deformation via direct absorption). The output signal is transduced through a transimpedance amplifier, demodulated by a lock-in amplifier at the 2f frequency, and analyzed by a personal computer. Simultaneously, a camera captures the laser profile for beam monitoring.

Supplementary Note 1. Discussion and comparison regarding the design and fabrication of tuning forks

The design and fabrication of tuning forks are strongly governed by the intrinsic material properties and the target application. The fabrication methods of representative tuning forks are summarized in Supplementary Table 1. Quartz tuning forks, typically produced by photolithography and wet chemical etching from Z-cut quartz wafers, exhibit excellent frequency stability, high mechanical Q-factor, and low temperature drift, making them ideal for precision sensing and timing applications. Silicon tuning forks, fabricated using photolithography and deep reactive ion etching (DRIE) within standard MEMS processes, offer high fabrication reproducibility and seamless compatibility with on-chip integration, however their lack of intrinsic piezoelectricity limits direct signal transduction. In contrast, $LiNbO_3$ tuning forks, fabricated by UV photolithography and optical-grade dicing, leverage lithium niobate's strong piezoelectric and electro-optic properties to enable efficient transduction and potential coupling with optical or acoustic fields. Nonetheless, their brittle crystalline nature and processing complexity impose strict requirements on machining precision. Polymer-based (PVDF) tuning fork, formed through film casting, mechanical shaping, and electrode deposition, provide a flexible, lightweight, and low-cost alternative. Despite their lower Q-factor and reduced frequency stability compared with crystalline counterparts, PVDF designs are advantageous for flexible, wearable, or chemically harsh sensing environments. Overall, the trade-off between precision, cost, mechanical robustness, and integration feasibility dictates the optimal material and fabrication strategy for a given tuning-fork application.

Supplementary Table 1 Design and fabrication method of existing tuning forks.

Materials	Piezoelectric	Fabrication method	S. Ref
$LiNBO_3$	yes	UV photolithography and optical grade dicing	This work
quartz	yes	photolithography and etching	[1]
silicon	no	photolithography and etching	[2]
polymer	yes	film casting and mechanical processing	[3]
aluminum	no	mechanical processing	[4]

S. Ref Supplementary References.

Supplementary Note 2. Design and simulation of LN-MFP

The resonance frequency and quality factor (Q) of the LN-MFP are governed by both material properties and geometric configuration. In this work, the geometry was carefully optimized to balance the competing requirements of its multifunctional operation, including PAS, LITES, and photodetection. To guide this optimization, we present a developed systematic design procedure detailing how the geometric parameters of the LN-MFP were selected and refined.

(1) Optimization for Resonance

The geometry and resonance of the LN-MFP were optimized by varying two key geometric parameters: the tine length l and width w, while keeping all other parameters fixed (see Supplementary Fig. 3a for definitions). A parametric finite-element sweep of l and w revealed that the fundamental resonance f_0 decreases monotonically with increasing l and with decreasing w (Supplementary Fig. 3b). In the simulated design space f_0 ranged from ~ 34.5 kHz (l =6 mm, w = 2 mm) to ~2.8 kHz (l = 18 mm, w =1 mm).

For the LN-MFP, excessively high resonant frequency f_0 yields shorter vibration period, limiting the time available for the device to accumulate acoustic energy. Since the light-source modulation must match the LN-MFP resonance frequency, high f_0 values require faster modulation rates, which reduce the efficiency of photoacoustic generation for molecules with long vibrational-to-translational (V–T) relaxation times. For example, CO_2 exhibits V–T relaxation times on the order of 100 μs (~ 10 kHz), and modulation beyond this frequency significantly diminishes photoacoustic generation efficiency. By contrast, very low f_0 increases susceptibility to low-frequency environmental disturbances such as mechanical vibration, air currents and $1/f$ (pink) noise.

Balancing these effects, an optimal frequency f_0 ~ 10.5 kHz was selected, corresponding to tine dimensions of l=11.5 mm, w=1.7 mm.

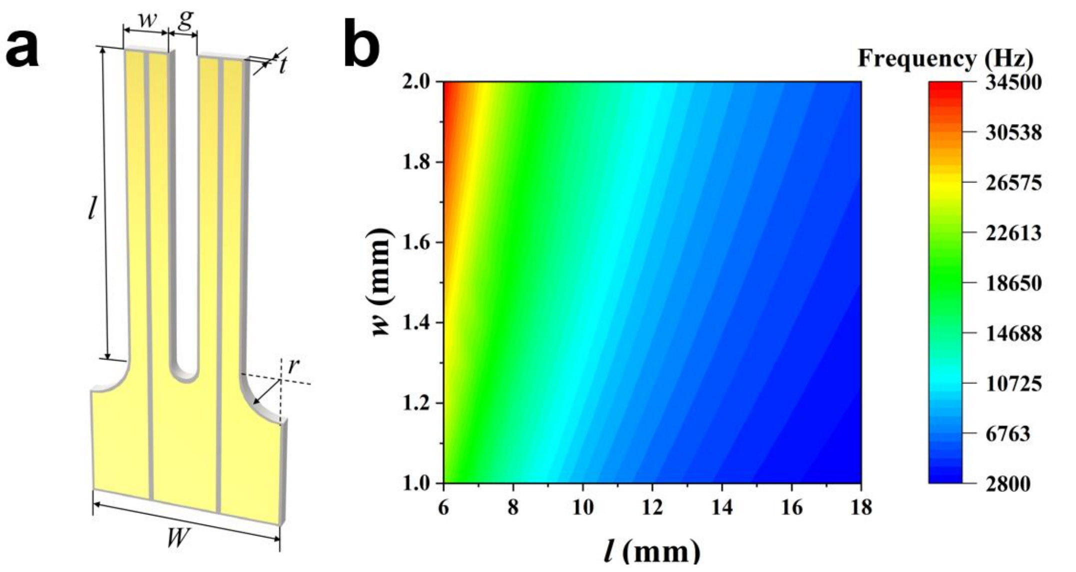

Supplementary Fig. 3 | Resonant frequency of the LN-MFP. a Schematic of the LN-MFP showing the defined geometric parameters: tine length *l*, base width *W*, tine width *w*, inter-tine gap *g*, thickness *t*, and fillet radius *r*. **b** Finite-element simulation of the fundamental resonant frequency f_0 as a function of tine length *l* and width *w*. The results show that f_0 decreases markedly with increasing *l* and with decreasing *w*; spanning a frequency range from ~34.5 kHz to ~2.8 kHz across the design space.

(2) Optimization for PAS

The LN-MFP geometry was optimized for photoacoustic spectroscopy (PAS) through coupled acoustic–structural finite-element simulations. In the acoustic excitation model, a cylindrical pressure source was positioned within the tine gap to reproduce the spatial distribution of the photoacoustic pressure wave and to induce symmetric tine oscillation (see Supplementary Fig. 4a). For each geometric configuration the total piezoelectric charge generated by the device was calculated.

The resulting contour map of normalized piezoelectric charge exhibits a clear and monotonic dependence on tine dimensions (Supplementary Fig. 4b). The PAS signal strength increases with greater tine length *l* and with smaller tine width *w*. Physically, longer and more slender tines exhibit larger tip displacements and stronger strain localization near the tine base under acoustic excitation, hereby generating a higher piezoelectric charge output and enhancing signal amplitude.

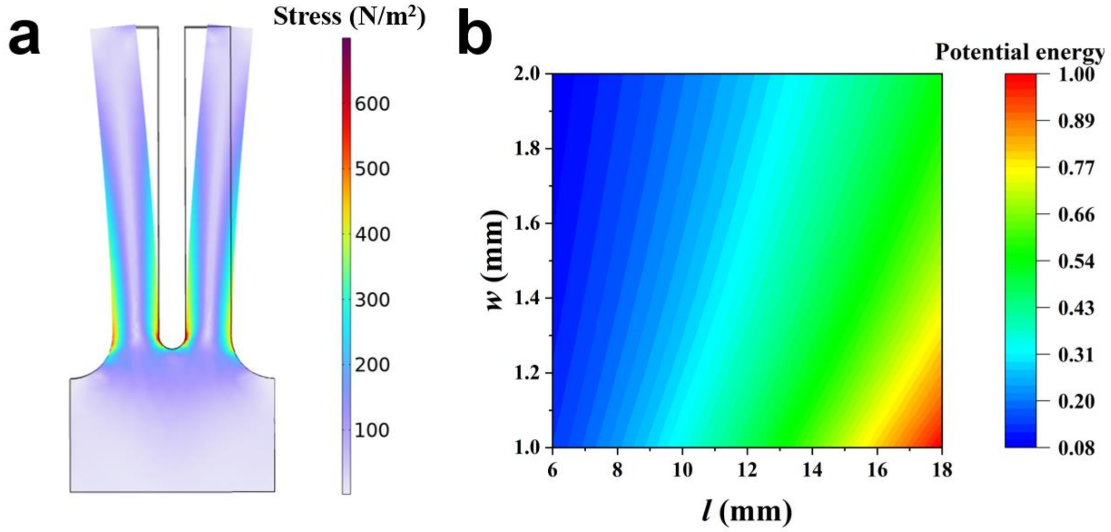

Supplementary Fig. 4 | Stress distribution and piezoelectric energy optimization of the LN-MFP. a Simulated deformation and strain distribution of the LN-MFP under PAS; **b** Normalized piezoelectric charge as a function of tine length *l* and tine width *w*.

(3) Optimization for LITES and photodetection

Because both LITES and direct photodetection originate from light-induced heating, they were analyzed within a unified thermoelastic framework. In this model, a localized heat source was applied at the LN-MFP base to simulate the photothermal excitation induced by incident light (see Supplementary Fig. 5a). For each geometric configuration, the total piezoelectric charge collected by the electrode was computed. The resulting contour map of normalized piezoelectric charge in (Supplementary Fig. 5b) reveals a systematic increase with the tine width *w* and only a weak dependence on tine length *l*. This trend arises from several factors. First, wider tines provide a larger heated volume and absorption area for a given illumination spot, leading to greater overall thermal expansion and a larger bending moment. Second, increasing *w* enlarges the electromechanically active volume and electrode-covered area, enhancing conversion of localized strain into piezoelectric charge. Third, because the thermoelastic excitation is concentrated near the base, variations in tine length *l* exert minimal influence on the thermal-to-mechanical coupling in this region.

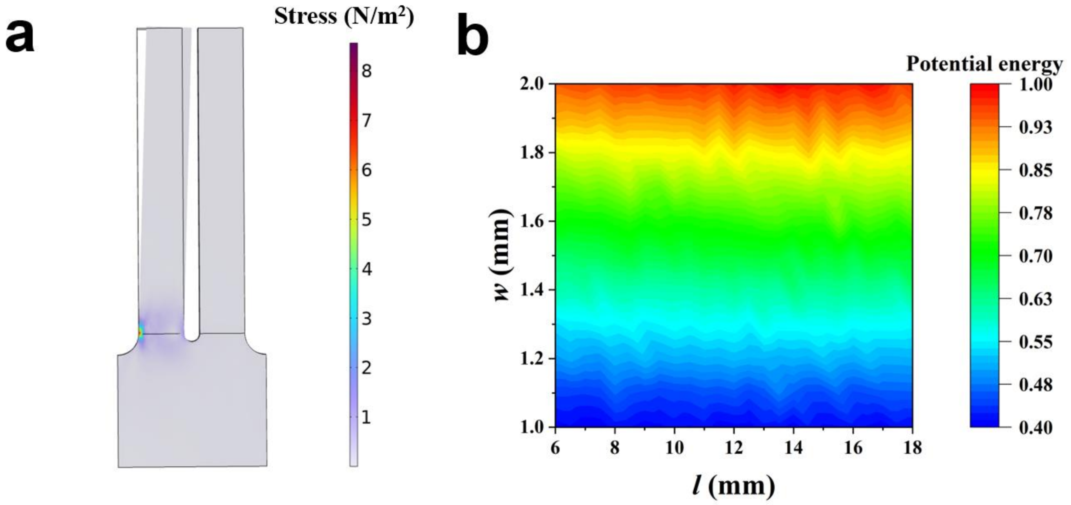

Supplementary Fig. 5 | Thermoelastic stress distribution and piezoelectric response optimization of the LN-MFP. a Simulated deformation and strain distribution of the LN-MFP under LITES and photodetection excitation; **b** Contour map of normalized piezoelectric charge as a function of tine length *l* and tine width *w*.

(4) Other geometry parameters

-Tine gap *g*

To accommodate diverse optical sources, from visible LEDs to mid-IR QCLs, and to balance optical and acoustic requirements, the tine gap was fixed at *g*=1.0 mm. The gap size is governed by two competing effects. For LITES and photodetection, light is incident on the outer tine surfaces, whereas in PAS operation, the excitation beam must traverse the gap without contacting the electrodes. If the gap is too narrow, the laser spot overlaps the metalized tines, introducing significant photothermal background noise. Conversely, if the gap is too wide, the acoustic pressure field weakens, reducing acoustic-to-mechanical coupling efficiency. Therefore, a 1.0 mm gap represents a practical compromise between these competing optical and acoustic constraints.

- Device thickness *t*

Commercial lithium-niobate wafers are typically available in thicknesses of ~500 μm. Within the explored design-space, variation in device thickness produced only minor effects on the fundamental resonance compared to the dominant influence of tine length and width. Its impact on electromechanical and thermoelastic responses was similarly limited. Considering factors such as wafer availability, fabrication precision,

mechanical robustness and process repeatability, a thickness of t=500 μm was selected for the fabricated LN-MFP devices.

(5) Conclusion

In summary, a comprehensive finite-element parameter sweep combined with multiphysics simulation was conducted to optimize the LN-MFP geometry across three key performance dimensions: resonant behavior, photoacoustic (PAS) response, and thermoelastic/photodetection response. The inherently opposing design requirements of PAS and LITES/photodetection necessitated a balanced configuration. The finalized structure, with l=11.5 mm, w =1.7 mm, g=1.0mm, and t=500 μm, exhibits a fundamental resonant frequency near 10.5 kHz. This optimized design achieves high PAS sensitivity alongside strong LITES/photodetection responsivity, ensuring multifunctional operation with excellent mechanical stability, fabrication compatibility and system integration compatibility.

Supplementary Note 3. Manufacture of the LN-MFP and PCB-level co-packaged LN-MFP sensor

(1) Manufacture of the LN-MFP

The LN-MFP device was fabricated on a double-side-polished Y+128°-cut $LiNbO_3$ wafer with a thickness of 500 μm. The process began with wafer slicing, edge grinding, and double-side polishing to ensure surface flatness and uniform thickness followed by ultrasonic cleaning to remove microscopic contaminants.

A Cr/Au (20/200 nm) bilayer was then deposited to form the electrode layer using an electron-beam evaporation system (model DE400, Texas Instruments, USA) under a base vacuum of 3×10^{-6} Torr. The Cr adhesion layer was evaporated at 30 W with a rate of 0.5 Å/s for 400 s, followed by deposition of the Au layer at 40 W, 0.8 Å/s for 2,500 s ensuring uniform film adhesion and high surface conductivity.

Subsequently, 355 nm UV photolithography was employed to define the electrode geometry directly on the wafer surface. Both electrode patterning and device dicing were performed using a UV laser (5 W, 20 kHz repetition rate, 30 μs pulse width), achieving maskless fabrication with ±10 μm precision. During electrode definition,

each exposure lasted 10 s, sufficient to pattern the Cr/Au electrodes. For dicing, the same laser system operated for over 1200 s, fully cutting through the 500 μm-thick LN substrate.

Following laser processing, an "optical-grade dicing" and polishing step was implemented to refine structural precision. The LN-MFPs were mounted on a UV-sensitive adhesive film attached to a stainless-steel polishing frame. A high-precision optical dicing system equipped with a 56 mm-diameter, 150 μm-thick resin-bonded diamond wheel was used to polish the sidewalls at a rotation speed of 10, 000 rpm and a feed rate of 0.2 mm/s, with deionized-water circulation maintaining a stable temperature of 18 °C.

Finally, the fabricated LN-MFP was integrated onto the substrate via gold wire bonding technology. The bonding process used controlled heat, pressure, and ultrasonic energy to connect the surface electrodes of the LN-MFP to the silicon carrier. The bonded gold wires were 20 μm in diameter and the backside electrode was attached with conductive silver paste to ensure reliable grounding and enhanced electrical contact.

(2) Fabrication of PCB-level co-packaged LN-MFP sensor

The fabrication process began with the design and preparation of a transimpedance preamplifier PCB board, which serves as the signal conditioning and amplification interface for the LN-MFP device. A gold-coated silicon substrate was then patterned using photolithography to define the electrode layout. To enable free vibration of the LN-MFP tines, a micro-machined recess was etched beneath the LN-MFP region on the silicon substrate, ensuring that the fork structure remained suspended and mechanically isolated from the base.

Subsequently, the processed silicon substrate was bonded to the preamplifier PCB, establishing the mechanical foundation for electrical integration. The LN-MFP was then attached to the silicon substrate using conductive silver paste, forming a robust electrical contact between the LN-MFP's bottom electrode and the silicon-based electrodes. In parallel, the QCL chip was mounted onto the same silicon substrate using a specialized adhesive or solder material, enabling optical alignment with the LN-MFP

region.

Finally, gold wire bonding was employed to complete the electrical interconnections. The surface electrodes of the LN-MFP and QCL chip were bonded to the silicon electrodes, which were in turn connected to the corresponding pads on the PCB. This integration process ensured precise electrical continuity, compact structural assembly, and efficient signal transmission, providing the foundation for a stable and miniaturized all-lithium-niobate spectroscopic chips.

Supplementary Note 4. Resonance profile of the LN-MFP

Each side of the LN-MFP is divided into three distinct regions: a central region where negative charges accumulate and two side regions where positive charges are concentrated. Due to the piezoelectric properties of lithium niobate, the charge distribution on the back of the LN-MFP mirrors that on the front side, with opposite polarities. To optimize signal extraction, the central electrode on the front side is electrically connected to the side electrodes on the back, forming one output terminal of the MFP. Conversely, the side electrodes on the front are linked to the central electrode on the back, creating the second output terminal. This electrode configuration effectively captures the differential charge distribution generated during device operation.

The LN-MFP's resonance characteristics were measured to evaluate its performance as a piezoelectric resonator. The device was electrically excited to induce mechanical vibration, generating an output charge proportional to the vibration amplitude. Supplementary Fig. 6 presents the recorded vibration amplitude of the LN-MFP. The resonance curve exhibits a distinct peak at the fundamental resonance frequency, confirming an efficient vibrational response. An electrical excitation signal of 40 mVpp was used to excite the LN-MFP. The LN-MFP has a frequency of 10,485 Hz and a Q factor of 1,621. This remarkable enhancement underscores the superior piezoelectric performance of lithium niobate in the proposed device configuration.

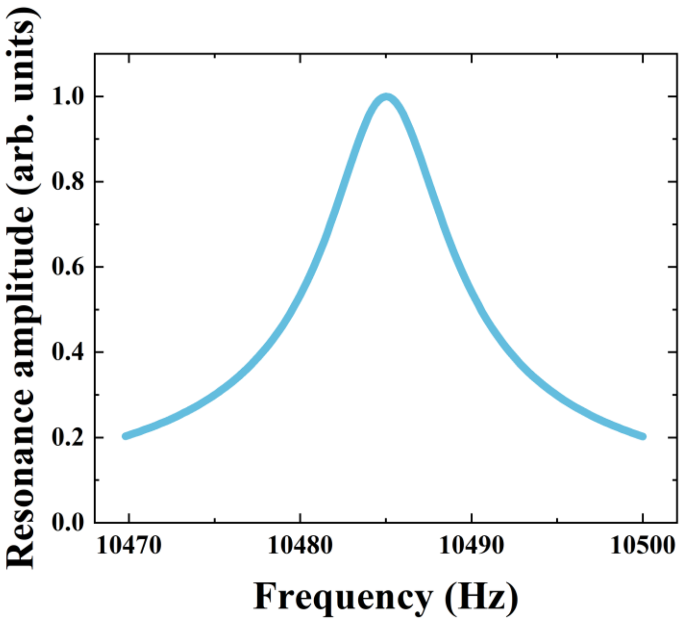

Supplementary Fig. 6 | Resonance profile of the LN-MFP as a function of excitation frequency. Measured resonance amplitude of the LN-MFP as a function of excitation frequency, showing a resonant frequency of 10,485 Hz and a Q factor of 1,621.

Supplementary Note 5. Optimization of photoacoustic detection

To optimize the LN-MFP for lithium niobate-enhanced photoacoustic spectroscopy detection, the effect of laser beam positioning between the tines and along their length was systematically studied. The laser spot was incrementally shifted from the top opening to the base of the tines, and the corresponding PAS signal amplitude was retrieved and modeled using finite element modeling (FEM) with COMSOL Multiphysics®. The results, shown in Supplementary Fig. 7a, indicate that when the laser beam was positioned approximately 2 mm from the top of the tines, the LN-MFP output reached its peak.

An acoustic resonator was incorporated at the 2 mm position to construct a spectrophone. The acoustic resonator consisted of two independent stainless steel capillary tubes, mounted perpendicular to the LN-MFP plane and aligned coaxially with the laser beam, as illustrated in Supplementary Fig. 7b. The gap between the capillaries ends and the LN-MFP plane was fixed at ~20 μm to ensure optimal coupling. We systematically tested various capillary lengths and measured the corresponding signal amplitude, identifying 13 mm as the optimal length. After optimization, the acoustic

resonator was designed with a length of 13 mm, an inner diameter of 2.6 mm, and an outer diameter of 3 mm. According to Supplementary Fig. 7c, the resonator shifted the system’ s resonance from 10,485 Hz to 10,488 Hz, indicating acoustic－mechanical coupling between the resonator and the LN-MFP. The quality factor decreased to ~1300 due to resonant coupling. Through resonant amplification of acoustic waves, the acoustic resonator enhanced the LN-MFP signal by 79 times, enabling detection of ultraweak acoustic signals.

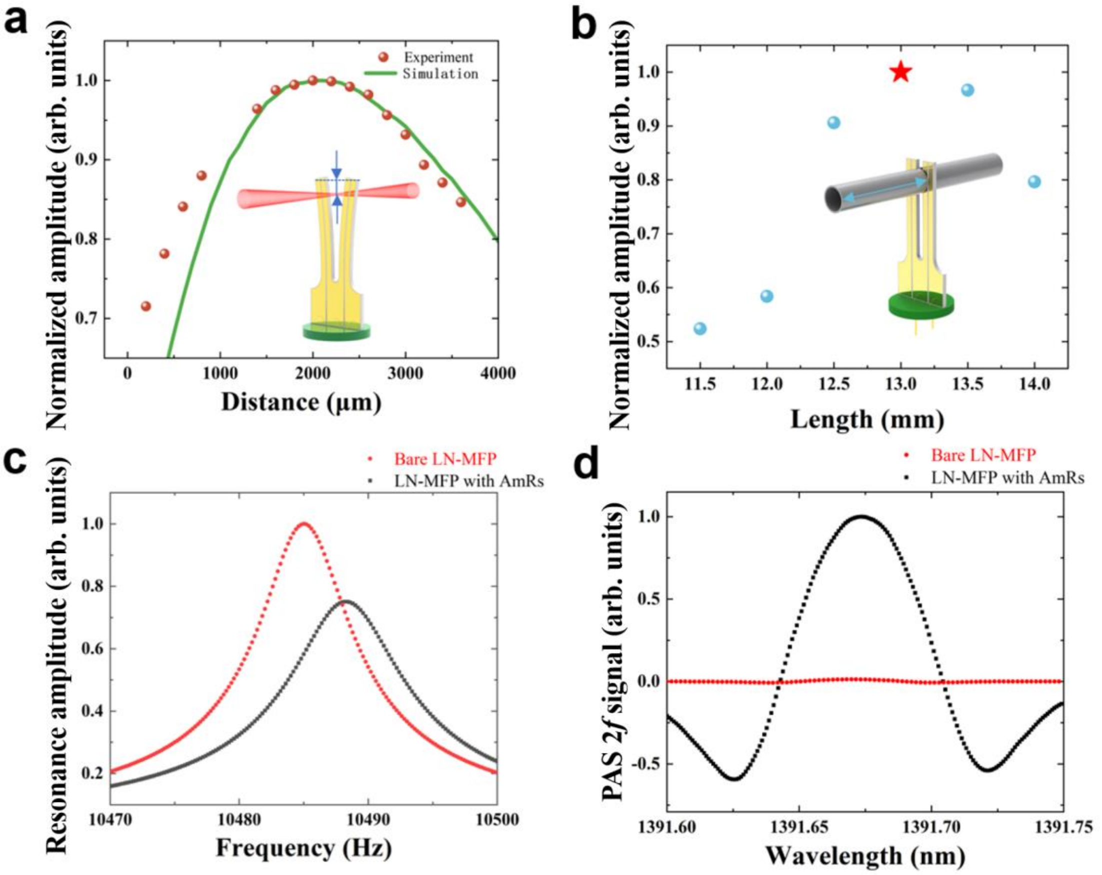

Supplementary Fig. 7 | The LN-MFP coupled with AmRs. a Simulated and measured PAS signals as a function of the laser beam position along the LN-MFP tines. **b** PAS signal amplitude as the function of an acoustic resonator’s length. **c** Resonance curves measured for a bare LN-MFP and a LN-MFP with an AmRs; **d** PAS 2*f* signal based on H_2O measurements when using a bare LN-MFP and a LN-MFP with an AmRs.

Supplementary Note 6. Optimization of thermoelastic detection

The 1.3 μm NIR DFB laser, previously described, was used to target the water absorption line at 7185.6 cm^{-1}. The position of light incidence on the tuning fork is a

crucial parameter, as the thermal deformation effect varies along the tines, directly influencing the LN-MFP's output response. As shown in Supplementary Fig. 8a, we experimentally compared the photodetection responses when the laser irradiated either the bottom side (black curve) or the front surface (red curve) of the LN-MFP tine. The results clearly indicate that side illumination produces a stronger signal, with a peak amplitude more than twice that of front illumination. We systematically investigated the impact of thermal excitation position on the LITES signal of the LN-MFP. Supplementary Fig. 8b presents the signal amplitude as a function of the laser beam position along the left tine. For symmetry, the same results are expected when exciting the right tuning fork prong. The results indicate that the optimal position for maximizing the thermoelastic effect is 10.5 mm from the top of the tine. This location aligns with the region where thermal-induced deformation most effectively couples with the mechanical resonance of the device.

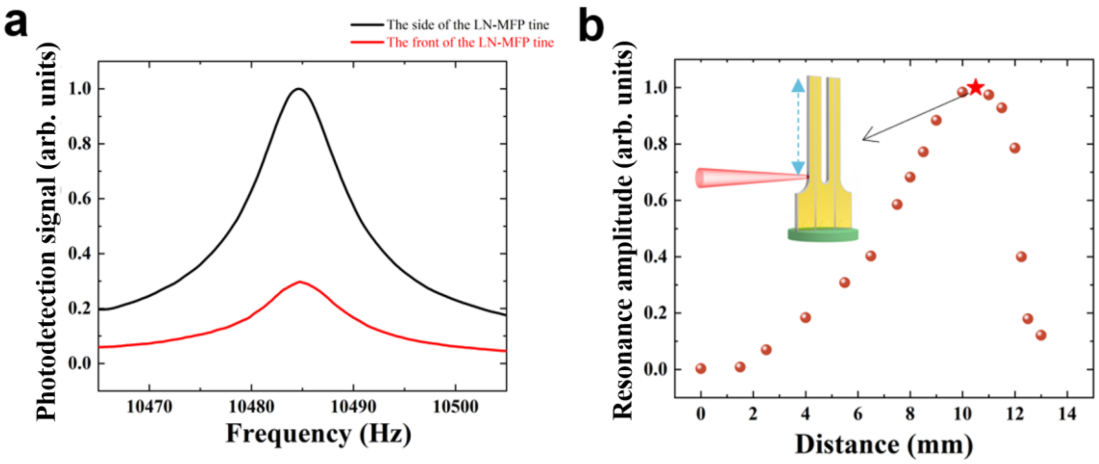

Supplementary Fig. 8 | Optimization of the laser incident position. a LITES signal of the LN-MFP as a function of the laser beam position along the left tuning fork tine**. b** Normalized photodetection signal measurements when laser was incident to the side and to the front of the LN-MFP tine surface.

Supplementary Note 7. Determination of minimum detection limit for light

The correlation between laser modulation depth and LN-MFP vibration amplitude was systematically analyzed. The modulation depth was progressively reduced toward zero, allowing for an accurate assessment of the device's photodetection capabilities. The

photodetection sensitivity S of the LN-MFP was calculated using the following equation:

$$S = \frac{P_i}{A_i} \cdot A_0 \quad (1)$$

where P_i represents the laser power at modulation depth i, and A_i and A_0 represent the vibration amplitudes at modulation depth i and zero, respectively.

Supplementary Note 8. PCB-level Co-packaged LN-MFP Sensor Module

(1) Optical-alignment and stability**.**

The QCL chip was aligned directly to the LN-MFP tine gap without external collimating optics. The QCL far-field characterized using a mid-IR camera, exhibited a divergence of ~70°. The tine gap, under an optical microscope was ~1.0 mm and the separation between QCL facet and LN-MFP was ~100 μm. The QCL chip was soldered to a silicon carrier, and after fine optical alignment under the microscope, the LN-MFP was secured in position with conductive silver paste. This free-space coupling configuration enables robust proof-of-concept PAS measurements with relaxed lateral alignment tolerances.

(2) Limitations and mitigation.

In the absence of beam-shaping optics, part of the emitted power falls outside the LN-MFP tine gap, leading to stray illumination of the tine surfaces. This produces photothermal background signals, reduces optical coupling efficiency, and can ultimately limit long-term stability and the achievable minimum detection limit (MDL). To enhance coupling efficiency and robustness in future iterations, several strategies will be explored: (i) integrating a micro-collimator or compact mid-IR lens between the QCL facet and the tine gap to reduce divergence and improve overlap; (ii) incorporating molded or etched microlenses on the silicon carrier to shape the beam at the chip level; (iii) implementing monolithic coupling approaches, such as integrated waveguide or butt-coupling to eliminate free-space tolerances and further stabilize the optical alignment.

Supplementary Note 9. The calculation of NNEA

To quantitatively assess the performance of the LN-MFP–based spectroscopic sensor, the normalized noise-equivalent absorption (NNEA) was evaluated for the different gas channels. The NNEA was calculated according to the following expression:

$$\mathrm{NNEA} = \frac{\alpha_{\min} P_{eff}}{\sqrt{ENBW}} \tag{2}$$

where P_{eff} is the effective optical power delivered to the sensing region, α_{min} denotes the minimum detectable absorption coefficient, and $ENBW$ is the equivalent noise bandwidth of the detection system. The $ENBW$ was determined by the settings of the lock-in amplifier. In our experiments, a time constant of 1 s and a filter slope of 12 dB/oct resulted in an equivalent noise bandwidth of 0.25 Hz.

Supplementary Note 10. Comparison of the performance of the LN-MFP with existing PAS, LITES, and pyroelectric sensors.

In photoacoustic spectroscopy, the key parameters that govern detection performance are the laser wavelength, optical power, and integration time. These parameters together with the MDL, relative MDL (accounting for the proportionality of the photoacoustic signal to laser power), and normalized noise equivalent absorption (NNEA) coefficient, are summarized in Supplementary Table 2. Notably, the LN-MFP sensor is PCB-scale offering inherent advantages in compactness, robustness, and ease of system integration.

Supplementary Table 2 | PAS detection of trace gases using LN-MFP and QTF

Target analyte	Detector type	Wavelength (μm)	IT (s)	Power (mW)	MDL (ppb)	RMDL (ppm W)	NNEA (cm^{-1} W $Hz^{-1/2}$)
NO_2	LN-MFP	0.45	1000	1200	2	2.4×10^{-3}	/
NO_2	QTF [7]	0.45	120	47	21	9.87×10^{-4}	/
H_2O	LN-MFP	1.39	6300	19	25	4.75×10^{-4}	5.82×10^{-9}
H_2O	QTF [8]	1.39	1	/	2000	/	1.2×10^{-8}
C_2H_2	LN-MFP	1.53	1000	3700	3.5	1.3×10^{-2}	2.7×10^{-8}
C_2H_2	QTF [9]	1.53	370	5	21	1.05×10^{-4}	/
CO_2	LN-MFP	2	1900	10.6	350	3.71×10^{-3}	2.53×10^{-8}
CO_2	QTF [10]	2	365	5	2640	1.32×10^{-2}	2.5×10^{-8}
CH_4	LN-MFP	3.3	1200	6.5	2.5	1.63×10^{-5}	8.36×10^{-9}

CH_4	QTF [11]	3.3	1	/	50	/	2.9×10^{-9}
NH_3	LN-MFP	9.7	1100	82.3	0.35	2.88×10^{-5}	6.58×10^{-10}

IT integration time, MDL minimum detection limit, RMDL relative minimum detection limit, NNEA normalized noise equivalent absorption coefficient.

LN-MFP lithium niobate as a multi-functional integrated platform, QTF quartz tuning fork.

LN-MFP's competitive photoacoustic performance arises from several complementary physical and practical factors. First, single-crystal lithium niobate exhibits substantially higher effective piezoelectric coupling (for appropriate crystal orientations and tensor components) than α-quartz, enabling $LiNbO_3$ devices to generate stronger electrical signals—and thus higher signal-to-noise ratios—for a given mechanical strain [5]. Second, the device geometry and resonant frequency were carefully optimized to balance acoustic coupling, thermoelastic drive and readout bandwidths ensuring robust multimodal transduction even for slowly relaxing species such as CO_2. Finally, lithium niobate's mature and rapidly evolving photonics ecosystem, featuring broad optical transparency, strong electro-optic properties and wafer-level processing, facilitates the integration of on-chip optics, waveguides and micro-collimation components that are challenging to implement in conventional quartz tuning-fork systems [6]. The monolithic LN-MFP also minimizes parasitic capacitance and interconnect losses, enabling compact low-noise electronics near the transducer, thereby lowering the noise floor and enhancing NNEA.

Supplementary Table 3 | Photodetector using LN-MFP and pyroelectric/photovoltaic sensors.

Detector type	Category	Characterized wavelength range	NEP (W/√Hz)	Response time	Responsivity (V/W)
LN-MFP	Pyroelectric	450−9770 nm	5.65×10^{-8}	1 s	373
QTF [12]	Pyroelectric	1540 nm	/	1 s	47.4
$LiNbO_3$[13]	Pyroelectric	/	1.62×10^{-7}	1.9 s	/
$LiNbO_3$[14]	Pyroelectric	8−11 μm	/	29 ms	/
$LiTaO_3$[15]	Pyroelectric	X-rap	5.02×10^{-8}	/	1800
$Sb_2Te_3-Bi_2Te_3$[16]	Pyroelectric	600−700 nm	8.0×10^{-9}	341 μs	38
HgCdTe [17]	Photovoltaic	4.8 μm	1.7×10^{-13}	13 ns	/
Graphene [18]	Photovoltaic	1550 nm	4.54×10^{-12}	/	210

LN-MFP lithium niobate as a multi-functional integrated platform, QTF quartz tuning fork.

NEP noise equivalent power.

A concise comparison of the LN-MFP with representative pyroelectric and tuning-fork detectors is provided in Supplementary Table 3. In the current prototype the LN-MFP demonstrates high responsivity (~373 V W^{-1}) and a noise-equivalent power on the order of 5.6×10^{-8} W $Hz^{-1/2}$; measured with a 1 s lock-in time constant (corresponding to ~0.25 Hz detection bandwidth). The device operates across a broad wavelength range from 450 nm to 9.77 μm and integrates PAS, LITES and direct photodetection functionalities on a single substrate. The reported performance reflects a deliberate, system-level optimization, balancing geometry and readout to meet the combined requirements of all three sensing modalities. Although the LN-MFP is not necessarily optimized for any single modality, it uniquely combines high per-power responsivity, multimodal functionality and compatibility with wafer-scale photonics. Several straightforward engineering routes remain to further improve the absolute MDL, NEP and bandwidth, including the use of low-noise transimpedance amplifiers, micro-collimation or waveguide coupling to enhance optical power delivery, and tailored absorptive coatings at the tine base. These improvements will be explored in future work to further enhance performance while preserving the platform's integration advantages.

Supplementary Table 4 | LITES detection of trace gases using LN-MFP and QTF.

Target analyte	Detector type	Wavelength (μm)	IT (s)	AL (cm)	MDL (ppm)	RMDL (ppm m)
NO_2	LN-MFP	0.45	450	2.5	1	2.5×10^{-2}
H_2O	LN-MFP	1.39	1600	2.5	2.5	6.25×10^{-2}
H_2O	QTF [19]	1.39	289	50	1.4	7×10^{-1}
C_2H_2	LN-MFP	1.53	1500	2.5	0.45	1.13×10^{-2}
C_2H_2	QTF [20]	1.53	70	20	0.36	7.2×10^{-2}
CO_2	LN-MFP	2	1200	2.5	35	8.75×10^{-1}
CO_2	QTF [21]	2	100	100	20	2×10^{1}
CH_4	LN-MFP	3.3	1900	2.5	0.9	2.25×10^{-2}
CH_4	QTF [22]	1.65	206	420	0.0035	1.47×10^{-2}
NH_3	LN-MFP	9.7	1200	2.5	1.2	3×10^{-2}

LN-MFP lithium niobate as a multi-functional integrated platform, QTF quartz tuning fork.

IT integration time, AL absorption length, MDL minimum detection limit, RMDL relative minimum detection limit.

For LITES, the key parameters include the laser wavelength, absorption length (AL), and integration time, as summarized in Supplementary Table 4. Since the MDL is directly proportional to the AL, the comparison includes not only the MDL but also the relative MDL for a fair and comprehensive evaluation. Supplementary Table 4 shows that the LN-MFP attains superior RMDL for most gas species (H_2O, C_2H_2, CO_2, NO_2, NH_3), with CH_4 as the only minor exception. This advantage stems from three factors: larger effective piezoelectric coupling of Y+128° $LiNbO_3$, an electrode layout that maximizes charge collection at regions of peak modal strain, and a resonance (~10.5 kHz) properly tuned to enhance thermoelastic/acoustic energy build-up for slowly relaxing species.

Supplementary Note 11. The light sources and other instruments used for LN-MFP experiment

Supplementary Fig. 9 shows a photo of the experimental setup used for the LN-MFP measurements. A function generator (Tektronix AFG3102) supplied the modulation waveform to the light sources. The sources, as listed in the Supplementary Table 5, were driven and temperature-stabilized by a current/TEC controller (Thorlabs ITC4005QCL). The emitted beam was routed through the detection module. A camera (Dataray WinCamD-IR-BB) was employed for beam profiling and alignment. The electrical output from the detection module was demodulated by a lock-in amplifier (SRS SR830). A personal computer was used for instrument control and data acquisition.

Supplementary Table 5. The wavelength and model of seven light sources.

Light source	Model	Wavelength (nm)	Power (mW)	Modulation method	IC (mA)	T (°C)
VIS LED	NICHIA 450	450	1200	AM	600	/
NIR LD	NTT Electronics Corporation	1390	19	FM	84	28
NIR LD	NTT Electronics Corporation	1536	3700*	FM	90	25
NIR LD	NTT Electronics Corporation	2004	10.6	FM	120	18
MWIR ICL	NANOPLUS	3370	6.5	FM	86	17

QCL Chip	Chinese Academy of Sciences	4590	14	FM	270	45
LWIR QCL	HEATHYPHOTON	9770	82.3	FM	420	20

VIS LED visible light emitting diode, NIR LD, MWIR ICL mid-wave infrared interband cascade laser, LWIR QCL long-wave infrared quantum cascade lasers, AM amplitude modulation, FM frequency modulation, *amplified by erbium-doped fiber amplifiers. IC: injection current. T temperature.

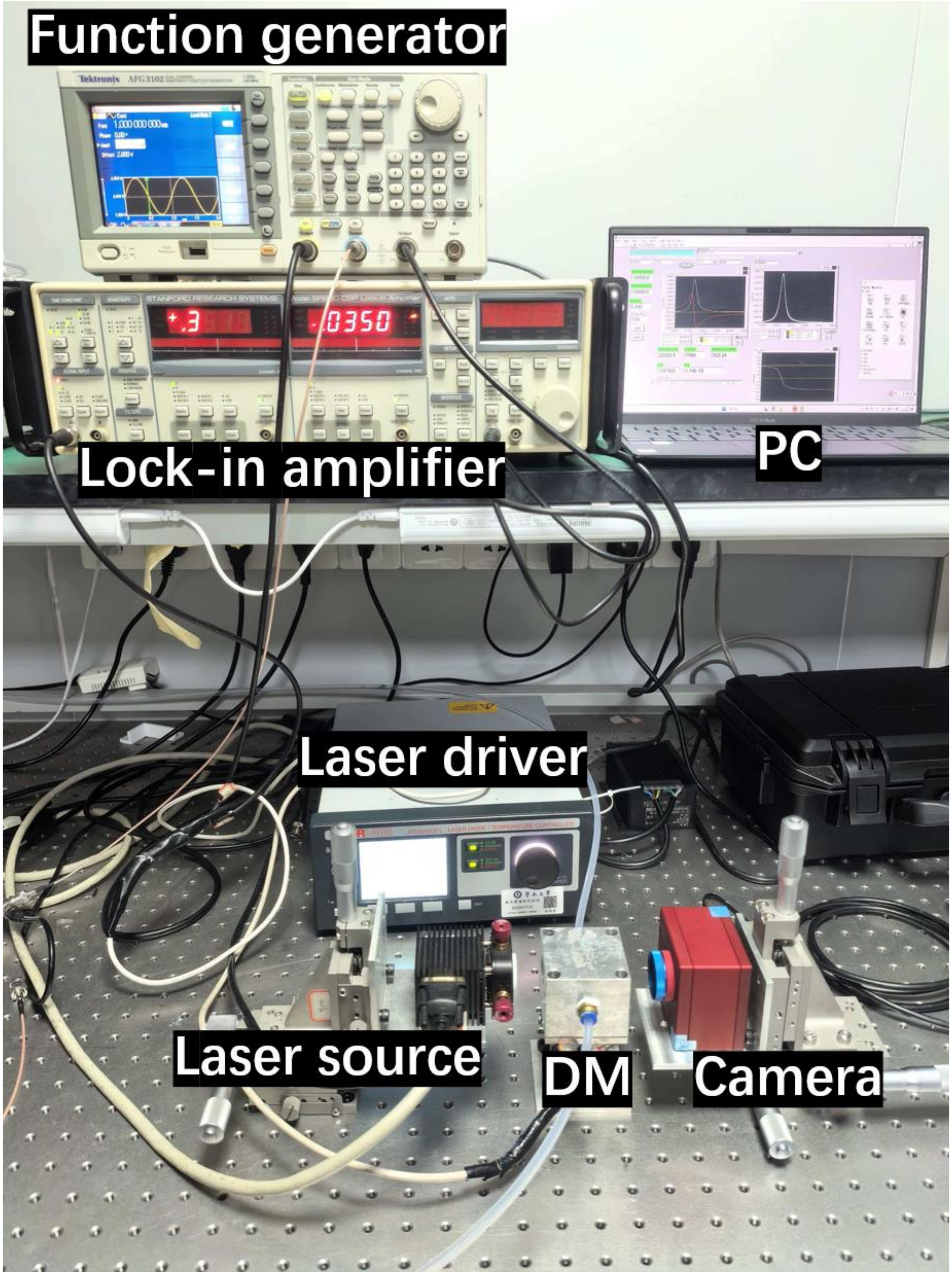

Supplementary Fig. 9 | Photo of the employed experimental setup. PC: personal computer, DM: detection module.

Data availability

The datasets generated and analysed during this study are available from the corresponding author upon reasonable request.

Supplementary References

1. Lin, H. et al. Application of standard and custom quartz tuning forks for quartz-enhanced photoacoustic spectroscopy gas sensing. *Appl. Spectrosc. Rev.* **58**, 562-584 (2023).
2. Lavrik, N. V. & Datskos., P. G. Optically read Coriolis vibratory gyroscope based on a silicon tuning fork. *Microsyst. Nanoeng.* **5**, 47 (2019).
3. Liu, K. et al. A novel photoacoustic spectroscopy gas sensor using a low cost polyvinylidene fluoride film. *Sens. Actuator B-Chem.* **277**, 571-575 (2018).
4. Pan, Y. et al. Miniaturized and highly-sensitive fiber-optic photoacoustic gas sensor based on an integrated tuning fork by mechanical processing with dual-prong differential measurement. *Photoacoustics* **34**, 100573 (2023).
5. Yue, W. et al. Crystal orientation dependence of piezoelectric properties in $LiNbO_3$ and $LiTaO_3$. *Opt. Mater.* **23**, 403-408 (2003).
6. Boes, A. et al. Lithium niobate photonics: Unlocking the electromagnetic spectrum. *Science* **379**, eabj4396 (2023).
7. Breitegger, P. et al. Towards low-cost QEPAS sensors for nitrogen dioxide detection. *Photoacoustics* **18**, 100169 (2020).
8. Rousseau R. et al. Monolithic double resonator for quartz enhanced photoacoustic spectroscopy. *Appl. Sci.* **11**: 2094 (2021).
9. Wang, R., Qiao, S., He, Y., & Ma, Y. Highly sensitive laser spectroscopy sensing based on a novel four-prong quartz tuning fork. *Opto-Electron. Adv.* **8**, 240275-1 (2025).
10. Zhang, Y. et al. Continuous real-time monitoring of carbon dioxide emitted from human skin by quartz-enhanced photoacoustic spectroscopy. *Photoacoustics* **30**, 100488 (2023).
11. Wu H. et al. Atmospheric CH_4 measurement near a landfill using an ICL-based QEPAS sensor with VT relaxation self-calibration. *Sens. Actuator B-Chem.* **297** 126753 (2019).
12. Zhou, S. et al. Realization of a infrared detector free of bandwidth limit based on quartz crystal tuning fork. *Opt. Laser Technol.* **113**, 261-265 (2019).
13. Aleks, M. et al. An overview of microelectronic infrared pyroelectric detector. *Eng. Sci.* **16**, 82-89 (2021).
14. Suen, J. Y. et al. Multifunctional metamaterial pyroelectric infrared detectors. *Optica* **4**, 276-279 (2017).
15. Kane, S. R. et al. Characterizing pyroelectric detectors for quantitative synchrotron radiation measurements. *Sensor. Actuat. A-Phys.* **387**, 116406 (2025).
16. Mauser, K. W. et al. Resonant thermoelectric nanophotonics. *Nat. Nanotechnol.* **12**, 770-775 (2017).
17. Wang, Y. et al. Fast uncooled mid-wavelength infrared photodetectors with heterostructures of van der Waals on epitaxial HgCdTe. *Adv. Mater.* **34**, 2107772 (2022).
18. Molaei-Yeznabad, A. & Abedi, K. Optimal design of graphene-based plasmonic enhanced photodetector using PSO. *Sci. Rep.* **14**, 15291 (2024).
19. Xu, L. et al. Multigas sensing technique based on quartz crystal tuning fork-enhanced laser

spectroscopy. *Anal. Chem.* **92** 14153-14163 (2020).

20. He, Y. et al. Hydrogen-enhanced light-induced thermoelastic spectroscopy sensing. *Photonics Res.* **13** 194-200 (2024).

21. Bojęś, P. et al. Dual-band light-induced thermoelastic spectroscopy utilizing an antiresonant hollow-core fiber-based gas absorption cell. *Appl. Phys. B* **129**, 177 (2023).

22. Shang, Z. et al. Robust and compact light-induced thermoelastic sensor for atmospheric methane detection based on a vacuum-sealed subminiature tuning fork. *Photoacoustics* **42**, 100691 (2025).
